# Staff perspectives for maintaining staff morale in a nursing home setting

**DOI:** 10.1002/alz.083712

**Published:** 2025-01-09

**Authors:** Dominique H. Como, Carin M. Wong, Victoria Shier, Cara A. Lekovitch, Felicia M Chew, Julie Britton, Natalie E Leland

**Affiliations:** ^1^ University of Pittsburgh, Pittsburgh, PA USA; ^2^ University of Southern California, Los Angeles, CA USA; ^3^ Thomas Jefferson University, Philadelphia, PA USA; ^4^ Genesis Healthcare, Wall, NJ USA

## Abstract

**Background:**

The COVID‐19 pandemic had a major impact on healthcare, contributing to a mass exodus of the workforce. This poses a concern for Alzheimer’s disease and related dementias (ADRD) care, which benefits from consistent care routine and staff that know the resident. Therefore, it is important to understand nursing home staff perspectives on maintaining high staff morale, which impacts recruitment, retention, and care quality.

**Method:**

Embedded within a larger pragmatic clinical trial evaluating two approaches to dementia care, nursing home staff (n = 327) were interviewed about their perspectives of dementia care before and after the emergence of COVID‐19. Purposive sampling was used to gain insights across different job roles (e.g., administrators, direct care providers, ancillary support). Staff morale surfaced during the interviews in the context of the evolving COVID‐19 pandemic and the competing demands on staff. Thematic analysis was used to analyze this emergent topic.

**Result:**

Nursing home staff identified several themes necessary to improve staff morale in light of the negative impact the pandemic had on the workforce striving to care for residents living with dementia. These themes reflected actions that are needed at the individual, interpersonal, and organizational level. The themes include (1) self‐care strategies, (2) peer‐to‐peer support, and (3) leadership approaches and policies. Self‐care strategies were described as being initiated by the staff member for the benefit of their own mental or physical wellbeing. Examples of self‐care included: stepping away from the situation, engaging in spiritual practices (e.g., prayer), and exercising to relieve stress. Descriptions of peer‐to‐peer support reflected instances where staff supported one‐another through teamwork, mentorship, and communication. Staff also highlighted how organizational leadership contributed to morale by ensuring staff felt appreciated and supported. Staff provided examples of leadership initiatives (e.g., scheduling, transparency, incentives) to convey support of staff.

**Conclusion:**

These findings highlight the actionable steps staff and administrators can take to foster staff morale among those who care for residents living with ADRD. Given the impact the pandemic had on the healthcare workforce and the challenge many staff who work on dementia units continue to face, staff morale may be a key factor in addressing staff retention and care quality.

